# Association of vancomycin-induced acute kidney injury with trough versus AUC monitoring in patients receiving extended durations of therapy

**DOI:** 10.1017/ash.2023.490

**Published:** 2023-12-04

**Authors:** C. Tyler Pitcock, Aric Schadler, David S. Burgess, Donna R. Burgess, Sarah E. Cotner, Jeremy Van Hoose, Eric R. Gregory, Katie L. Wallace

**Affiliations:** 1 Department of Pharmacy Services, University of Kentucky HealthCare, Lexington, KY, USA; 2 Department of Pharmacy Practice and Science, University of Kentucky College of Pharmacy, Lexington, KY, USA; 3 Department of Pharmacy Services, The University of Kansas Health System, Kansas City, KS, USA; 4 Department of Pediatrics, Kentucky Children’s Hospital, Lexington, KY, USA

## Abstract

**Objective:**

Vancomycin therapy is associated with an increased risk of acute kidney injury (AKI). Previous studies suggest that area under the curve (AUC) monitoring reduces the risk of AKI, but literature is lacking to support this in patients receiving longer durations of vancomycin therapy.

**Design:**

Retrospective cohort study.

**Method:**

Patients ≥18 years old, admitted between August 2015 and July 2017 or October 2017 and September 2019, and received at least 14 days of intravenous (IV) vancomycin therapy were included in the study. Our primary outcome was the incidence of AKI between trough monitoring and AUC monitoring groups using Kidney Disease Improving Global Outcomes criteria. Secondary outcomes included inpatient mortality, median inpatient length of stay, and median intensive care unit length of stay.

**Results:**

Overall, 582 patients were included in the study, with 318 patients included in the trough monitoring group and 264 included in the AUC monitoring group. The median duration of vancomycin therapy was 23 days (interquartile range, 16–39). Patients within the trough monitoring group had a higher incidence of AKI compared to the AUC monitoring group (45.6% vs 28.4%, *p* < 0.001). Furthermore, logistic regression analysis showed that AUC monitoring was associated with a 54% lower incidence of AKI (OR 0.46, 95% CI [0.31–0.69]). All-cause inpatient mortality was numerically higher in the trough monitoring group (12.9% vs 8.3%, *p* = 0.078).

**Conclusions:**

In patients who received at least 14 days of IV vancomycin therapy, AUC monitoring was associated with a lower incidence of AKI.

## Background

Vancomycin, a common antibiotic used to treat staphylococcal, streptococcal, and enterococcal infections, has been widely known to cause nephrotoxicity.^1–4^ Historically, trough goals of 15–20 mg/L have been used as a surrogate marker for an area under the curve (AUC) goal of 400–600 mg*h/L based on the 2009 consensus guidelines from Infectious Diseases Society of America, American Society of Health-System Pharmacists, and Society of Infectious Diseases Pharmacists. This therapeutic drug monitoring strategy was developed from data that illustrated that targeting troughs of ≥15 mg/L would have 90% probability of attaining an AUC goal of ≥400 mg*h/L when treating *Staphylococcus aureus* isolates that have a minimum inhibitory concentration of 1 mg/L using Monte Carlo simulation.^5^ Subsequent studies found that targeting troughs of 15–20 mg/L increased the risk of patients developing nephrotoxicity.^6,7^ Furthermore, it was found that targeting troughs of 15–20 mg/L may underestimate the true AUC by 25%^7^; however, these studies did not look specifically at clinical outcomes to assess whether this had an impact. In 2020, the same committee group released new guidelines that recommended AUC-guided dosing and monitoring for patients receiving vancomycin as opposed to trough monitoring.^8^ Primary literature has not demonstrated consistent improvement in clinical outcomes with AUC monitoring but has shown improved safety as a result of decreased nephrotoxicity. For this reason, the authors of the 2020 vancomycin guidelines supported AUC monitoring over trough. Additional studies have concluded that AUC-guided dosing and monitoring reduces the nephrotoxicity risk and institutional costs as a result.^9–15^

Unfortunately, there is no literature that analyzes the clinical impact of this dosing strategy in patients receiving longer durations of intravenous (IV) vancomycin therapy. The purpose of this study was to analyze the impact of trough versus AUC monitoring on acute kidney injury (AKI) in patients receiving at least 2 weeks of vancomycin therapy.

## Methods

### Study design

This was a retrospective cohort study evaluating University of Kentucky Health Care (UKHC) patients between August 1st, 2015 and September 12th, 2019 who received at least 14 days of IV vancomycin therapy. Data were collected through the University of Kentucky Center for Clinical and Translational Science Enterprise Data Trust (CCTS). Our study was approved by the Institutional Review Board of the University of Kentucky.

### Eligibility criteria

Patients 18 years of age or older who were admitted to UKHC, received a minimum duration of 14 days of inpatient IV vancomycin therapy, and had vancomycin serum concentration(s) drawn within 96 hours of initiation between August 2015 to July 2017 and October 2017 to September 2019 were included in our analysis. Per UKHC guidelines, serum concentration(s) were drawn around the 4th or 5th dose of vancomycin to reflect steady state. The UKHC implemented vancomycin AUC monitoring in September 2017. Prior to 2017, the UKHC monitored vancomycin using only trough concentrations. Patients who received IV vancomycin therapy from August 1st, 2015 to July 31st, 2017 were placed in the trough monitoring cohort, while those who received therapy from October 1st, 2017 to September 12th, 2019 were placed in the AUC monitoring cohort. The gap between cohort dates was used to address any delay in transitioning to new standard of care procedures at UKHC. Both trough and AUC groups were required to have a trough serum concentration drawn within 2 hours prior to administration of an IV vancomycin dose, and patients in the AUC monitoring group had to have a random serum concentration drawn within 2–5 hours after administration of a vancomycin dose. Patients were excluded if their creatinine clearance was ≤30 ml/min using the corrected Cockcroft–Gault equation at the time of initiation,^16^ had a diagnosis of cystic fibrosis, history of kidney transplant, chronic kidney disease stage 3–5, or were pregnant. Patients who developed AKI during their hospital admission before initiation of vancomycin, within 48 hours of initiation of vancomycin, or 7 or more days after vancomycin was discontinued were also excluded as AKI was unlikely to be caused by vancomycin in these patients.

### Outcomes

The primary outcome was the incidence of AKI based on the Kidney Disease Improving Global Outcomes (KDIGO) Clinical Practice Guidelines^17^ definition using the change in serum creatinine from baseline. We excluded the urine output criteria and presence of continuous renal replacement therapy in our calculation of AKI. The initial serum creatinine was defined as the serum creatinine concentration observed directly prior to the initiation of vancomycin therapy. The first available serum creatinine value was used if no serum creatinine value was available prior to initiation of vancomycin. The highest serum creatinine was defined as the maximum serum creatinine between 48 hours of vancomycin initiation and up to 7 days after discontinuation of vancomycin therapy. Patients were classified as not having an AKI if the change in serum creatinine concentrations did not meet KDIGO criteria.

Secondary outcomes included median intensive care unit (ICU) length of stay, all-cause inpatient mortality (defined as death during hospitalization or transferred to hospice), median length of stay, median daily dose of vancomycin, and median vancomycin trough concentration. Patient demographics that were collected included age, weight, sex, height, comorbidities, baseline serum creatinine, days of concomitant nephrotoxic medication use, length of hospitalization, and the number of concomitant nephrotoxic medications that were administered during the patient’s hospital stay. Nephrotoxic medications that were analyzed included: aminoglycosides, angiotensin-converting enzyme inhibitors, angiotensin receptor blockers, vasopressors, IV contrast dye, acyclovir, valacyclovir, amphotericin B, loop diuretics, tenofovir disoproxil-containing medications, calcineurin inhibitors, sulfamethoxazole-trimethoprim, foscarnet, polymyxin B, colistimethate, and nonsteroidal anti-inflammatory drugs.

### Statistical analysis

Patient demographics were analyzed using a Student’s *t*-test or Mann–Whitney U test for parametric and nonparametric continuous variables, respectively. Pearson’s chi-square was used for categorical variables that included five or more patients. Fisher’s exact test was used for categorical variables that included less than five patients. Medians with interquartile ranges (IQRs) were used for nonparametric results while means with standard deviations were used for demographics with approximately normal distributions. For categorical variables, counts and percentages were reported. A multivariable logistic regression was then completed using a backward elimination selection strategy to identify variables that were the primary drivers for explaining AKI incidence. Variables that were included in the regression model were race, days of concomitant nephrotoxin utilization, BMI ≥30, age, duration of vancomycin therapy, weight, gender, dose of vancomycin, trough concentrations ≥15 mg/L, creatinine clearance, total daily vancomycin dose, history of kidney dysfunction, AUC monitoring, number of concomitant nephrotoxins, length of hospital stay, Charlson comorbidity index stage, and ICU admission.

## Results

### Baseline characteristics

**Figure 1.**
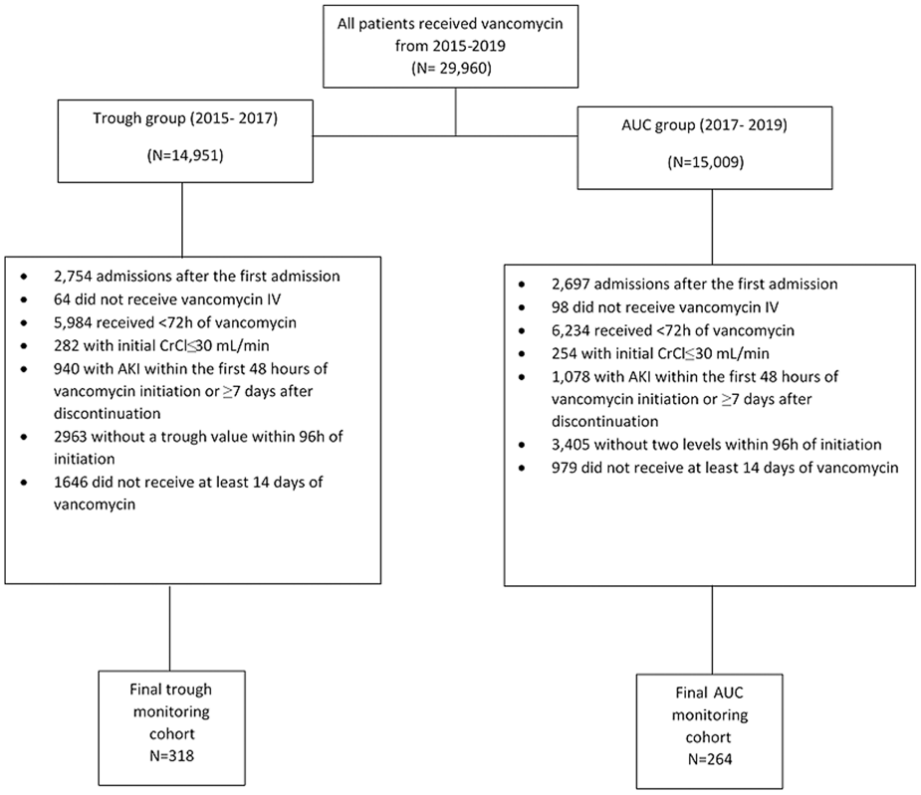
Flowchart of exclusion criteria.

Overall, 582 patients were included in the study with 318 patients in the trough monitoring group and 264 patients in the AUC monitoring group (Figure 1). Patients had a median age of 46 years old (IQR, 35–59) (Table 1). The majority of our patients were Caucasian (94.3%) and male (58.2%). There were no statistically significant differences between trough monitoring and AUC monitoring groups with the exception of one comorbidity. The trough monitoring group had a lower incidence of patients with diabetes mellitus with complications (6.3% vs 17.4%, *p* < 0.001). Overall, 91.8% of our patients were on concomitant nephrotoxic medications at the time of initiation of IV vancomycin therapy. Additionally, patients were on a median of two concomitant nephrotoxins (IQR, 1–3) in both trough monitoring and AUC monitoring groups. Approximately 47% of the patient population were admitted to the ICU at the time of initiation of IV vancomycin therapy.

**Table 1.** Baseline characteristics, comorbidities, and baseline laboratory concentrations

	Overall	Trough	AUC	*p*-value
	*N* = 582	*N* = 318	*N* = 264	
Age^a^ (years)	46 [35–59]	47 [35–60]	43 [35–59]	0.441
Gender^b^ (male)	339 (58.2)	187 (58.8)	152 (57.6)	0.765
Race^b^ (Caucasian)	549 (94.3)	305 (95.9)	244 (92.4)	0.070
ICU^b^	273 (46.9)	152 (47.8)	121 (45.8)	0.636
Weight^a,^ ^d^ (kg)	77.1 [63.6–92.3]	77.1 [63.6–93.0]	77.0 [63.9–92.3]	0.549
Body mass index^a,^ ^d^ (kg/m^2^)	26 [22–31]	26 [22–30]	26 [23–31]	0.770
Albumin^c,^ ^d^ (g/dL)	2.49 ± 0.68	2.53 ± 0.71	2.43 ± 0.66	0.128
Bilirubin^a,^ ^d^ (mg/dL)	0.5 [0.3–0.9]	0.5 [0.4–0.9]	0.5 [0.3–0.9]	0.395
Serum creatinine^a^ (mg/dL)	0.77 [0.61–0.99]	0.76 [0.60–1.01]	0.78 [0.63–0.97]	0.697
Creatinine clearance^c,^ ^d^ (ml/min)	121.4 ± 59.2	115.5 ± 45.3	116.8 ± 42.6	0.726
Incidence of aminoglycoside use^b^	85 (14.6)	53 (16.7)	32 (12.1)	0.122
Incidence of IV contrast use^b^	156 (26.8)	81 (25.5)	75 (28.4)	0.426
Number of concomitant nephrotoxins^a^	2 [1–3]	2 [1–3]	2 [1–3]	0.246
Days of concomitant nephrotoxins^a^	13 [4–25]	14 [4–22]	13 [4–28]	0.482
Incidence of concomitant nephrotoxin use^b^	534 (91.8)	292 (91.8)	242 (91.7)	0.945
Charlson comorbidity index^b,^ ^d^	Stage 1 (mild ≤2): 269 (46.7)	Stage 1 (mild ≤2): 147 (46.2)	Stage 1 (mild ≤2): 122 (47.3)	0.087
	Stage 2 (moderate 3–4): 141 (24.5)	Stage 2 (moderate 3–4): 69 (21.7)	Stage 2 (moderate 3–4): 72 (27.9)	
	Stage 3 (severe ≥5): 166 (28.8)	Stage 3 (severe ≥5): 102 (32.1)	Stage 3 (severe ≥5): 64 (24.8)	
History of congestive heart failure^b,^ ^d^	85 (14.8)	53 (16.7)	32 (12.4)	0.151
History of diabetes mellitus without complications^b,^ ^d^	133 (23.1)	64 (20.1)	69 (26.7)	0.061
History of diabetes mellitus with complications^b,^ ^d^	65 (11.3)	20 (6.3)	45 (17.4)	<0.001
History of stage 1 and 2 chronic kidney disease^b,^ ^d^	31 (5.4)	22 (6.9)	9 (3.5)	0.070

Note. AUC, area under the curve; ICU, intensive care unit; HIV, human immunodeficiency virus; AIDS, acquired immunodeficiency syndrome; IQR, interquartile range; SD, standard deviation.

a Median (IQR).

b Number (%).

c Mean ± SD.

d *n* = 504 for BMI, *n* = 494 for albumin, *n* = 459 for bilirubin, *n* = 582 for creatinine clearance, *n* = 576 for Charlson comorbidity index, *n* = 576 for history of congestive heart failure, history of diabetes mellitus without complications, history of diabetes mellitus with complications, and history of stage 1 and 2 chronic kidney disease.

### Outcomes

The incidence of AKI was 45.6% in the trough monitoring group and 28.4% in the AUC monitoring group (*p* < 0.001). Additionally, we observed a statistically significant difference between each individual stage of KDIGO (Table 2). In each stage, there was a consistently higher incidence of AKI in the trough monitoring group compared to the AUC monitoring group (stage 1: 22.0% vs 15.9%, stage 2: 10.7% vs 7.6%, and stage 3: 12.9% vs 4.9%, *p* < 0.001).

**Table 2.** Primary and secondary outcomes

	Overall	Trough	AUC	*p*-value
	*N* = 582	*N* = 318	*N* = 264	
Acute kidney injury^b^	220 (37.8)	145 (45.6)	75 (28.4)	<0.001
KDIGO stage 1 AKI^b^	112 (19.2)	70 (22.0)	42 (15.9)	<0.001
KDIGO stage 2 AKI^b^	54 (9.3)	34 (10.7)	20 (7.6)	<0.001
KDIGO stage 3 AKI^b^	54 (9.3)	41 (12.9)	13 (4.9)	<0.001
Trough concentration^a^ (mg/L)	11.7 [8.2–16.9]	11.5 [8.0–17.0]	11.8 [8.5–16.9]	0.288
Peak concentration^a^ (mg/L)			26.5 [21–32.6]	
Patient-specific area under the curve^a^ (AUC)			476.1 [383.8–612.1]	
Total daily dose of vancomycin^a,^ ^c^ (mg)	2500 [2000–3000]	2500 [2000–3000]	2500 [2000–3000]	0.566
Duration of therapy^a^ (days)	23 [16–39]	22 [16–40]	23 [16–39]	0.554
Inpatient mortality, all cause^b^	63 (10.8)	41 (12.9)	22 (8.3)	0.078
ICU length of stay^a,^ ^c^ (days)	12 [5–21]	13 [6–24]	10 [3–19]	0.008
Length of stay^a,^ ^c^ (days)	31 [20–45]	34 [21–46]	29 [19–45]	0.058
Loading dose ≥ 20 mg/kg^b^	241 (41.4)	107 (33.6)	134 (50.8)	<0.001

Note. AUC, area under the curve; ICU, intensive care unit; IQR, interquartile range; AKI, acute kidney injury; KDIGO, Kidney Disease Improving Global Outcomes.

a Median (IQR).

b Number (%).

c *n* = 578 for total daily dose of vancomycin, *n* = 273 for ICU length of stay, and *n* = 574 for length of stay.

Patients within the trough monitoring group had a statistically significant longer ICU length of stay compared to the AUC monitoring group (13 days [6–24] vs 10 days [3–19], *p* = 0.008) (Table 2). Less patients received a loading dose greater than or equal to 20 mg/kg in the trough monitoring group compared to AUC monitoring group (33.6% vs 50.8%, *p* < 0.001). The median AUC within the AUC group was 476.1 (IQR, 383.8–612.1). There was no statistically significant difference found in trough concentrations between the two groups (11.5 mg/L [8–17.0 mg/L] in the trough group vs 11.8 mg/L [8.5–16.9 mg/L] in the AUC group, *p* = 0.288).

### Multivariable logistic regression model

A multivariable regression analysis was conducted using the variables previously mentioned. AUC monitoring, number of concomitant nephrotoxins used, overall hospital length of stay, ICU admission, and moderate and severe Charlson comorbidity stages were found to be statistically significant after using the backwards elimination method (Table 3). AUC monitoring was associated with a 54% lower incidence (OR 0.46, 95% CI [0.31–0.69], *p* = <0.001) in AKI using KDIGO criteria.

**Table 3.** Multivariable regression model with respect to incidence of acute kidney injury

	Odds ratio	95% CI	*p*-value
AUC monitoring	0.46	0.31–0.69	<0.001
Inpatient length of stay	1.01	1.00–1.03	0.019
Number of concomitant nephrotoxins used	1.30	1.10–1.53	0.002
ICU admission	1.65	1.10–2.49	0.016
Charlson comorbidity stage 3	2.17	1.34–3.52	0.001
Charlson comorbidity stage 2	2.30	1.39–3.82	0.001

*Note*. CI, confidence interval; AUC, area under the curve; ICU, intensive care unit.

## Discussion

To the authors’ knowledge, this is the first study to analyze the incidence of AKI in patients receiving longer durations of vancomycin therapy. Our retrospective cohort study demonstrated that AUC monitoring of IV vancomycin was associated with a lower incidence of AKI compared to trough monitoring. After using a multivariate regression analysis to correct for confounders, AUC monitoring was associated with an over 50% decrease in AKI compared to trough monitoring. This closely resembles previous studies that analyzed the association of AKI with trough versus AUC monitoring. D’ Amico and colleagues^12^ looked at the incidence of AKI in patients with obesity using trough versus AUC monitoring and identified similar findings with a 36% lower incidence of AKI with AUC monitoring. Finch and colleagues^6^ also observed a lower incidence of AKI when using AUC monitoring after correcting for other confounders using cox proportional regression (HR 0.53, 95% CI [0.35–0.78], *p* = 0.002) and logistic regression models (OR 0.52, 95% CI [0.34–0.80], *p* = 0.003).

Additionally, Finch and colleagues^6^ found that AUC monitoring was associated with lower vancomycin total daily doses, lower AUC values, and lower trough concentrations, which all are believed to be associated with vancomycin-induced AKI.^2,18–20^ We did not observe a difference in total daily doses of vancomycin or trough concentrations between trough and AUC groups. It is unclear why these outcomes were similar between the two. This may be attributable to patients having augmented renal clearance, which is a common manifestation in critically ill patients.^21^ This seems possible given that the median trough concentration between the two groups was found to be around 12 mg/L, which is lower than the previously recommended trough goal of 15–20 mg/L.^5^

There was a difference between the two groups in the incidence of patients receiving loading doses ≥20 mg/kg with less patients in the trough monitoring group receiving them compared to the AUC monitoring group. Vancomycin monitoring education was provided during the implementation of AUC monitoring within our institution that included discussions regarding when to use loading doses, which could have led to the increased incidence of loading doses in our AUC monitoring patients. There is concern with using loading doses for vancomycin therapy due to the thought that higher total daily doses increase the risk of developing AKI.^2^ Research has not supported this concern but has demonstrated a therapeutic benefit in providing a loading dose.^22–25^ Our study adds to this evidence given that we did not find this to be an independent risk factor in developing AKI in our multivariate regression analysis.

Although our study provides additional data to support AUC monitoring, it is not without limitations. Given the retrospective study design and data retrieval from a large database, we observed some data points that were missing from patient charts within the electronic medical record (EMR). This can be seen in Tables 1 and 2 where we were not able to obtain all records of patients’ total daily doses, past medical history, and other patient specific laboratory values, although the number missing was minimal in comparison to the overall sample size. We also had to eliminate a few values for patient weight, height, and BMI due to incorrect imputation into the EMR. Furthermore, our patient population was a unique subset of patients who received a minimum of 14 days of inpatient vancomycin therapy. The onset of vancomycin-induced AKI typically occurs between 4 and 8 days of therapy.^20^ It could be possible that the patients within our study were less likely to develop AKI while on vancomycin therapy because they had not developed AKI early in their treatment regimen. This possibility could give rise to survivorship bias. Additionally, outcomes related to “time to AKI” and “site of infection” were outside of the scope of our study and should be considered limitations.

Overall, AUC monitoring was associated with a lower incidence of AKI compared to trough monitoring in patients receiving longer durations of vancomycin therapy. There was no observed benefit in mortality in patients monitored using AUC. Consideration should be given to implementing vancomycin AUC monitoring as a standard of practice to improve safety for patients receiving extended duration of therapy.
